# Genetic diversity of merozoite surface protein-1 C-terminal 42 kDa of *Plasmodium falciparum* (PfMSP-1_42_) may be greater than previously known in global isolates

**DOI:** 10.1186/s13071-018-3027-x

**Published:** 2018-08-06

**Authors:** Thị Lam Thái, Hojong Jun, Jinyoung Lee, Jung-Mi Kang, Hương Giang Lê, Khin Lin, Kyaw Zin Thant, Woon-Mok Sohn, Tong-Soo Kim, Byoung-Kuk Na

**Affiliations:** 1 Department of Parasitology and Tropical Medicine, and Institute of Health Sciences, Gyeongsang National University College of Medicine, Jinju, 52727 Republic of Korea; 2 BK21Plus Team for Anti-aging Biotechnology and Industry, Department of Convergence Medical Science, Gyeongsang National University, Jinju, 52727 Republic of Korea; 3 Department of Tropical Medicine, and Inha Research Institute for Medical Sciences, Inha University College of Medicine, Incheon, 22212 Republic of Korea; 4 Department of Medical Research Pyin Oo Lwin Branch, Pyin Oo Lwin, Myanmar; 5 Department of Medical Research, Ministry of Health and Sports, Yangon, Myanmar

**Keywords:** *Plasmodium falciparum*, PfMSP-1_42_, Myanmar, Polymorphism, Natural selection

## Abstract

**Background:**

The C-terminal 42 kDa region of merozoite surface protein-1 of *Plasmodium falciparum* (PfMSP-1_42_) is the target of an immune response. It has been recognised as one of the promising candidate antigens for a blood-stage malaria vaccine. Genetic structure of PfMSP-1_42_ has been considered to be largely conserved in the *P. falciparum* population. However, only limited information is currently available. This study aimed to analyse genetic diversity and the effect of natural selection on PfMSP-1_42_ among the Myanmar *P. falciparum* population and compare them with publicly available PfMSP-1_42_ from global *P. falciparum* populations.

**Methods:**

A total of 69 *P. falciparum* clinical isolates collected from Myanmar malaria patients in Upper Myanmar in 2015 were used. The PfMSP-1_42_ region was amplified by polymerase chain reaction, cloned and sequenced. Genetic structure and natural selection of this region were analysed using MEGA4 and DnaSP programs. Polymorphic nature and natural selection in global PfMSP-1_42_ were also investigated.

**Results:**

All three allele types (MAD20, K1, and RO33) of PfMSP-1_42_ were identified in Myanmar isolates of *P. falciparum*. Myanmar PfMSP-1_42_ displayed genetic diversity. Most polymorphisms were scattered in blocks 16 and 17. Polymorphisms observed in Myanmar PfMSP-1_42_ showed a similar pattern to those of global PfMSP-1_42_; however, they were not identical to each other. Genetic diversity of Myanmar PfMSP-1_42_ was relatively lower than that of PfMSP-1_42_ from different geographical regions. Evidence of natural selection and recombination were found. Comparative analysis of genetic polymorphism and natural selection in the global PfMSP-1_42_ population suggested that this region was not tightly conserved in global PfMSP-1_42_ as previously thought and is under the complicated influence of natural selection and recombination.

**Conclusions:**

Global PfMSP-1_42_ revealed limited, but non-negligible, genetic diversity by allele types and geographical origins. Complicated natural selection and potential recombination might have occurred in global PfMSP-1_42_. Comprehensive monitoring of genetic diversity for global PfMSP-1_42_ would be needed to better understand the polymorphic nature and evolutionary aspect of PfMSP-1_42_ in the global *P. falciparum* population. More thought would be necessary for designing a vaccine based on PfMSP-1_42_.

**Electronic supplementary material:**

The online version of this article (10.1186/s13071-018-3027-x) contains supplementary material, which is available to authorized users.

## Background

Malaria is one of the most important health burdens worldwide, especially in tropical and subtropical regions. It results in approximately 216 million clinical cases and about 450,000 deaths annually [1]. Despite many decades of intense study and efforts to control or eliminate malaria, it remains a significant global health problem. Currently, there is no commercially available malaria vaccine. Therefore, developing an effective vaccine is urgently needed to combat this disease. Among human-infecting malaria parasites, *Plasmodium falciparum* is the most medically important malaria parasite that is responsible for most malaria-related deaths globally [1]. However, due to the complexity of this parasite’s life-cycle and genetic variations of major vaccine candidate antigens, developing an effective malaria vaccine is still a difficult challenge.

Merozoite surface protein-1 (MSP-1) is a 195 kDa major surface glycoprotein of merozoites. This protein binds to human erythrocytes in a sialic acid-dependent manner. It plays a pivotal role in erythrocyte invasion by merozoites [2, 3]. MSP-1 is synthesised as a large precursor form which subsequently undergoes two independent proteolytic cleavage events. First, the large precursor protein is cleaved into four polypeptides (83, 30, 38 and 42 kDa) that form non-covalently associated complex. The 42 kDa fragment (MSP-1_42_) is further cleaved into 33 kDa (MSP-1_33_) and 19 kDa (MSP-1_19_) fragments when merozoites invades erythrocyte [3–5]. Only MSP-1_19_ remains on the merozoite surface and enters into erythrocyte whereas the others are released from the merozoite [6]. MSP-1 is highly immunogenic. Species-specific natural immune responses against this antigen have been reported in patients naturally exposed to *P. falciparum* and *P. vivax* [7–10]. In particular, the C-terminal region of MSP-1, MSP-1_19_ or MSP-1_42_, is of particular interest since individuals naturally infected with malaria acquire humoral immune responses against this domain [11–15]. Antibodies that recognise the C-terminal 42 kDa region of *P. falciparum* MSP-1 (PfMSP-1_42_) can inhibit the growth of the parasite or invasion of the merozoite into host erythrocytes [16–19]. Antibodies that react to PfMSP-1_42_ are associated with protection against *P. falciparum* infection in endemic settings [20, 21]. Both PfMSP-1_19_ and PfMSP-1_42_ have shown potential as subunit vaccines in animal models by conferring protection against infections [17, 22–25]. These results suggest that PfMSP-1_42_ is a promising candidate antigen for blood stage vaccine development.

Similar to other *P. falciparum* antigens, PfMSP-1 also shows polymorphism due to different mechanisms, including single nucleotide polymorphisms (SNPs), insertion/deletion of tandem repeats, and meiotic recombination [26, 27]. PfMSP-1 is divided into 17 distinct blocks that encode conserved, semiconserved, or variable regions of the protein [26, 27]. Among the variable regions, block 2 is the most polymorphic region in which repeat length polymorphisms are commonly identified. Size polymorphisms of this region between alleles enable this region as a molecular marker for parasite genotyping [28]. Therefore, the polymorphic nature of PfMSP-1 block 2 in global isolates has been extensively studied [29–37]. Meanwhile, PfMSP-1_42_ has been considered to be broadly conserved in different alleles of *P. falciparum* [26, 27]. However, only limited information for genetic diversity for this region is currently available. Genetic diversity analysis of MSP-1_42_ in small numbers of *P. falciparum* and *P. vivax* isolates suggests natural selection may be acting on the area and natural selection is an important factor in the maintenance and generation of genetic polymorphism observed in MSP-1_42_ [38]. Considering that PfMSP-1_42_ is a leading candidate for vaccine development, in-depth understanding of the polymorphic nature of this region would be beneficial to design optimised vaccine formulation. In this study, genetic diversity and natural selection of PfMSP-1_42_ in the Myanmar *P. falciparum* population were analysed. Data from this study were also compared with PfMSP-1_42_ sequences available for *P. falciparum* isolates from other malaria-endemic countries to gain insights into genetic polymorphism and natural selection of PfMSP-1_42_ in the global population(s).

## Methods

### Study areas and blood samples

A total of 69 blood samples collected from *Plasmodium falciparum*-infected Myanmar malaria patients were used. The patients enrolled in the study attended the public health centres in towns and villages of Naung Cho, Pyin Oo Lwin and Tha Beik Kyin townships in Upper Myanmar in 2015 with clinical symptoms of malaria. Malaria transmission in these areas was heterogeneous and seasonal with most clinical cases reported during the rainy season. *Plasmodium falciparum* infection was confirmed by Giemsa-stained thick and thin blood smear examination. Finger-prick blood samples were collected from the *P. falciparum*-infected patients before drug treatment. *Plasmodium falciparum* infection was further confirmed by polymerase chain reaction (PCR) targeting *18S* ribosomal RNA (rRNA) gene [39, 40]. The mean age of patients who donated the blood samples was 32.7 years-old and ranged between 13–57 years. Written consent was obtained from each patient before blood collection.

### Amplification and sequencing analysis of Myanmar PfMSP-1_42_

**Fig. 1:**
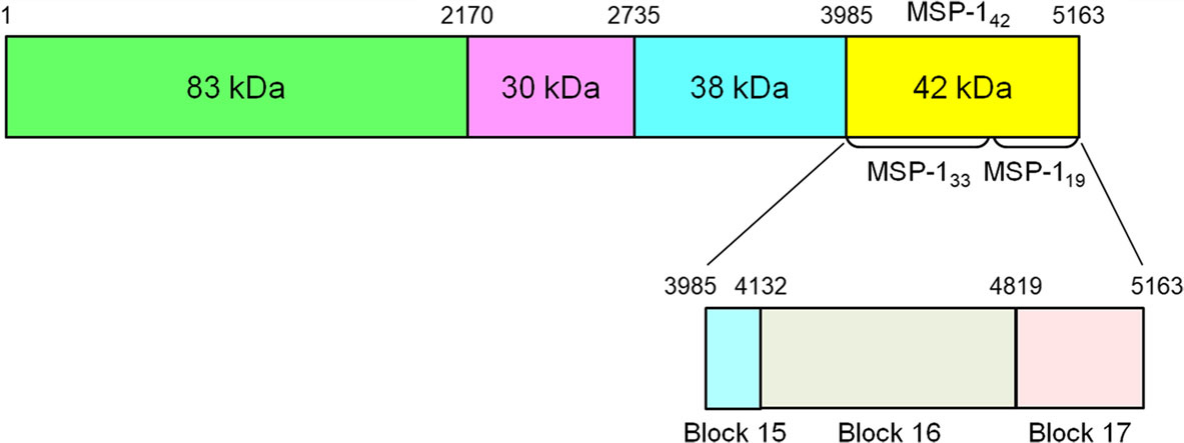
Schematic structure of PfMSP1. PfMSP1 is a large precursor protein that cleaved into four polypeptides (83, 30, 38 and 42 kDa). The 42 kDa fragment (MSP-1_42_) is further cleaved into 33 kDa (MSP-1_33_) and 19 kDa (MSP-1_19_) fragments when merozoite invades erythrocyte. In this study, the full-length PfMSP-1_42_ fragment flanking nucleotide positions from 3915 to 5163 in 3D7 sequence (XM_001352134) was amplified by a nested PCR. The numbers of nucleotide indicate coordinates according to the 3D7 sequence (XM_001352134)

Parasite genomic DNA was extracted from the blood filters using a QIAamp Blood Kit (Qiagen, Valencia, CA, USA). A nested PCR amplified the full-length PfMSP-1_42_ region flanking nucleotide positions from 3915 to 5163 in 3D7 sequence (XM_001352134) (Fig. 1). The oligonucleotide primers for the first round PCR were 5'-GAA ACT GTA TAA TAT TAA CAT GAG-3' and 5'-GAT AAT GAG GAA TAT TTA GAT CAA-3'. The primers for nested PCR were 5'-TTA CCC ATT TTT GGA GAA TCC GA-3' and 5'-TTA AGG TAA CAT ATT TTA ACT CCT-3'. The cycling condition for both amplification reactions was 95 °C for 10 min, followed by 30 cycles for 95 °C for 1 min, 53 °C for 1 min, and 72 °C for 1.5 min, and one cycle of the final extension at 72 °C for 10 min. Ex *Taq* DNA polymerase (Takara, Otsu, Japan) was used in all PCR steps to minimize the nucleotide misincorporation during PCR amplification. Each PCR product was analysed on 1.2% agarose gel, purified from the gel, and then ligated into T&A cloning vector (Real Biotech Corporation, Banqiao City, Taiwan). Each ligation mixture was transformed into *Escherichia coli* DH5α competent cells, and positive clones with the appropriate insert were selected by colony PCR with the nested primers. The cloned gene sequences were analysed by automatic nucleotide sequencing. To verify the sequences, at least two clones from each isolate were sequenced in both directions. Some isolates underwent three or four-fold sequence coverage to confirm the existence of rare polymorphisms. The nucleotide and deduced amino acid sequences of PfMSP-1_42_ were analysed using EditSeq and SeqMan in the DNASTAR package (DNASTAR, Madison, WI, USA). The nucleotide sequences obtained in this study have been deposited in the GenBank database under the accession numbers MF943252-MF943320.

### Sequence analysis and neutrality test

Nucleotide sequence polymorphism analysis was performed on the 69 Myanmar PfMSP-1_42_ sequences. The number of segregating sites (S), haplotypes (H), haplotype diversity (Hd), nucleotide diversity (π), and the average number of pairwise nucleotide differences within the population (*K*) were estimated using the DnaSP ver. 5.10.00 [41]. The π value was calculated to estimate the stepwise diversity throughout the entire PfMSP-1_42_ based on a sliding window of 100 bases with a step size of 25 bp. The rates of synonymous (dS) and non-synonymous (dN) substitutions were estimated and were compared with the *Z* test (*P* < 0.05) in MEGA4 program [42] using Nei & Gojobori’s method [43] with the Jukes-Cantor correction. To evaluate the neutral theory of evolution, the Tajima’s D [44] value and Fu and Li’s D and F statistics [45] were analysed using the DnaSP ver. 5.10.00 [41]. Positive values for Nei-Gojobori (dN-dS), Tajima’s D, and the Fu and Li’s D and F tests correspond to positive natural selection, whereas negative values correspond to negative or purifying selection.

### Population diversity of PfMSP-1_42_ among global *P. falciparum* isolates

Population diversity of PfMSP-1_42_ in global *P. falciparum* isolates was analysed. The PfMSP-1_42_ sequences from Thailand (*n* = 74, AB276006-AB276018, AB502546-AB502586 and D13343-D13363), Philippines (*n* = 43, AB502587-AB502628 and AB715434), Papua New Guinea (PNG: *n* = 77, AB502629-AB502704 and X05624), Solomon Islands (*n* = 41, AB502705-AB502745), Vanuatu (*n* = 91, AB715435-AB715519 and AB116596-AB116601), Ghana (*n* = 33, AB502514-AB502545 and AB276005), and Tanzania (*n* = 71 , AB502443-AB502513) were included in this study to analyse genetic diversity in global PfMSP-1_42_. Nucleotide diversity and the effect of natural selection for global PfMSP-1_42_ were analysed with the same methods described above. Recombination parameter (R), which included the effective population size and probability of recombination between adjacent nucleotides per generation, and minimum number of recombination events (Rm) were analysed by DnaSP ver. 5.10.00 [41].

### Haplotype network analysis

To investigate relationships among PfMSP-1_42_ haplotypes, the haplotype network for a total of 499 global PfMSP-1_42_ sequences including 69 Myanmar sequences and the 430 publicly available sequences of global isolates deposited in the GenBank database listed above was constructed using the program NETWORK version 5.0.0.3 with the Median Joining algorithm [46].

## Results

### Sequence polymorphism in Myanmar PfMSP-1_42_

Sequence analysis of 69 Myanmar PfMSP-1_42_ showed three different allele types (MAD20, K1, and RO33) in the Myanmar *P. falciparum* population. The MAD20 allele type was the most prevalent (37/69, 53.6%), followed by K1 (18/69, 26.1%), and RO33 (14/69, 20.3%) allele types. Sequence analysis of MAD20 allele types revealed a total of 21 distinct haplotypes in Myanmar sequences (Additional file 1: Figure S1). When compared to MAD20 (X05624) reference sequence, 19 SNPs were identified in MAD20 allele of Myanmar PfMSP-1_42_, including five synonymous and 14 non-synonymous SNPs. These non-synonymous SNPs caused di-morphic amino acid changes at 14 different positions. Most amino acid changes were found in block 16 and block 17 regions: seven amino acid changes (E105G, F183Y, D220N, F242L, T245H, S269N and Q290P) in block 16 and six amino acid changes (E307Q, T341A, T354K, S363N, R364G and L379F) in block 17. Meanwhile, only one amino acid change (I54F) was identified in block 15. These results suggest that blocks 16 and 17 show polymorphic characters in MAD20 allele types of Myanmar PfMSP-1_42_, which might contribute to inner allele diversity. For K1 allele types, a total of six distinct haplotypes were identified in Myanmar PfMSP-1_42_ sequences. Three synonymous and four non-synonymous SNPs were observed in these sequences when compared to K1 (X03371) reference sequence (Additional file 2: Figure S2). These four non-synonymous SNPs resulted in four di-morphic amino acid changes: E21G in block 15, R116G and H249R in block 16 and Q297E in block 17. For RO33 allele type, six different haplotypes were identified in Myanmar PfMSP-1_42_. When these sequences were compared to RO33 (Z35326) reference sequence, one tri-morphic (P244H/T) and eight di-morphic amino acid changes (F54I, L204I, D219N, Y245N, Q306E, K353T, N362S and G363R) were found in RO33 allele types of 14 Myanmar PfMSP-1_42_ sequences (Additional file 3: Figure S3). All amino acid changes except one (F54I) were observed in blocks 16 and 17. Collectively, sequence analysis of 69 Myanmar PfMSP-1_42_ indicated that most amino acid changes were mainly distributed in blocks 16 and 17 in all three allele types of Myanmar PfMSP-1_42_. All cysteine residues in block 17 were tightly conserved in all allele types of Myanmar PfMSP-1_42_. No insertion or deletion was detected in these sequences. Among 27 amino acid changes identified in these three allele types of Myanmar PfMSP-1_42_, 18 have been previously reported. However, the other nine substitutions (E105G, F242L, S269N, Q290P and T341A in MAD20 allele types; E21G, R116G and H249R in K1 allele types; L204I in RO33 allele types) were novel ones that have not been reported hitherto.

### Comparative analysis of amino acid polymorphisms between Myanmar PfMSP-1_42_ and global PfMSP-1_42_

**Fig. 2:**
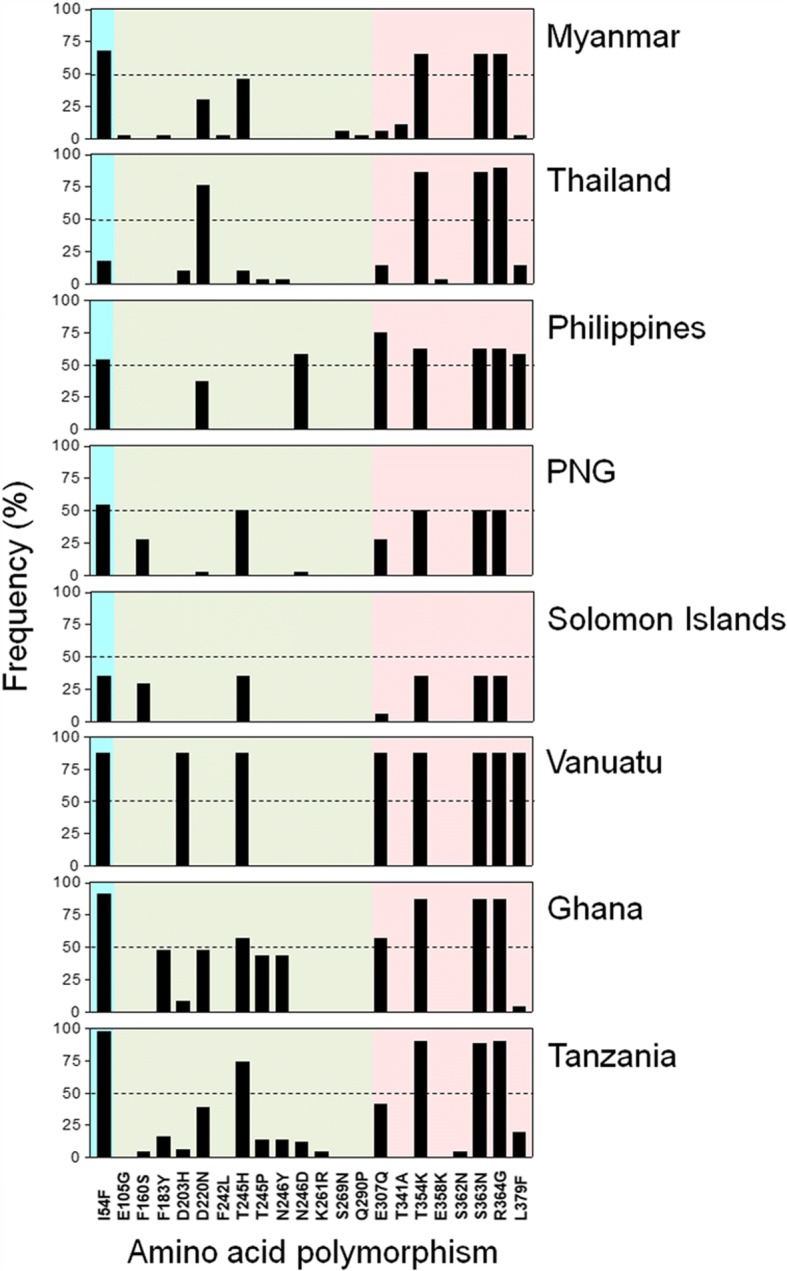
Comparison of amino acid polymorphisms in MAD20 allele types of PfMSP-1_42_ among global *Plasmodium falciparum* isolates. Positions and frequencies of amino acid changes found in MAD20 allele types of PfMSP-1_42_ in global *P. falciparum* isolates are compared. Blocks are indicated by different colors: block 15 (sky blue), block 16 (green), block 17 (pink). *Abbreviation*: PNG, Papua New Guinea

Overall patterns of amino acid polymorphisms identified in each allele type (MAD20, K1 and RO33) of Myanmar PfMSP-1_42_ were similar to those of global PfMSP-1_42_. However, they differed slightly from each other. MAD20 allele types of Myanmar PfMSP-1_42_ showed a different pattern of amino acid polymorphism compared to those from other geographical regions (Fig. 2). Compared to MAD20 (X05624) sequence, a total of 22 amino acid polymorphisms were identified in global PfMSP-1_42_. Their frequencies and distributions differed by country. Most amino acid polymorphisms in Myanmar and global PfMSP-1_42_ were mainly found in blocks 16 and 17. Five amino acid changes (I54F, E307Q, T354K, S363N and R364G) were commonly identified in all global PfMSP-1_42_ analysed in this study, although their frequencies were different by country or geographic area. I54F, the only amino acid change identified in block 15, was more prevalent in African (Ghana and Tanzania) PfMSP-1_42_ than that in other origins. Frequencies of F183Y and T245P were relatively high in African PfMSP-1_42_, but they were low or not detected in Asian (Myanmar, Thailand and Philippines) or the Pacific (PNG, Solomon Islands, and Vanuatu) PfMSP-1_42_. D220N was not identified or very rarely identified in Pacific PfMSP-1_42_.

**Fig. 3:**
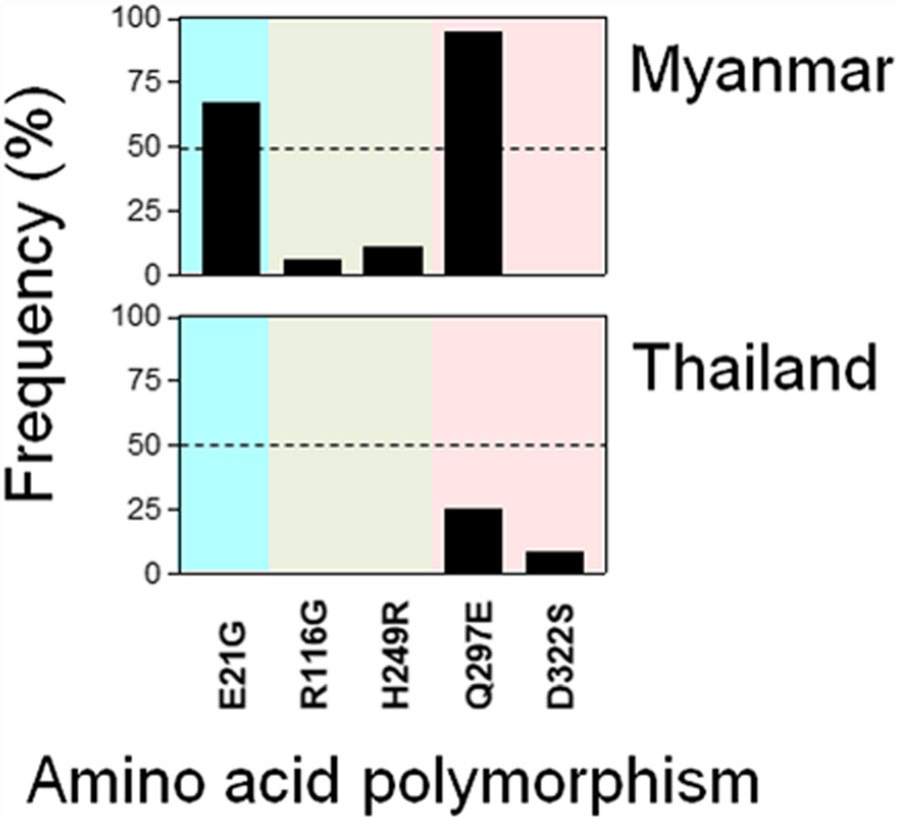
Comparison of amino acid polymorphisms in K1 allele types of PfMSP-1_42_ among global *Plasmodium falciparum* isolates. Positions and frequencies of amino acid changes found in K1 allele types of PfMSP-1_42_ in Myanmar and Thailand *P. falciparum* isolates are compared. Blocks are indicated by different colors: block 15 (sky blue), block 16 (green), block 17 (pink)

In the case of K1 allele type, only Thailand sequences have been reported so far. Overall amino acid change patterns of K1 allele types of PfMSP-1_42_ from Myanmar and Thailand were not similar (Fig. 3). E21G, R116G and H249R were identified only in Myanmar PfMSP-1_42_, although frequencies of R116G and H249R were low. Meanwhile, D322S was found just in Thailand PfMSP-1_42_. Q297E was identified in both Myanmar and Thailand PfMSP-1_42_, but its frequency was higher in Myanmar (94.4%) than in Thailand (25.0%).

**Fig. 4:**
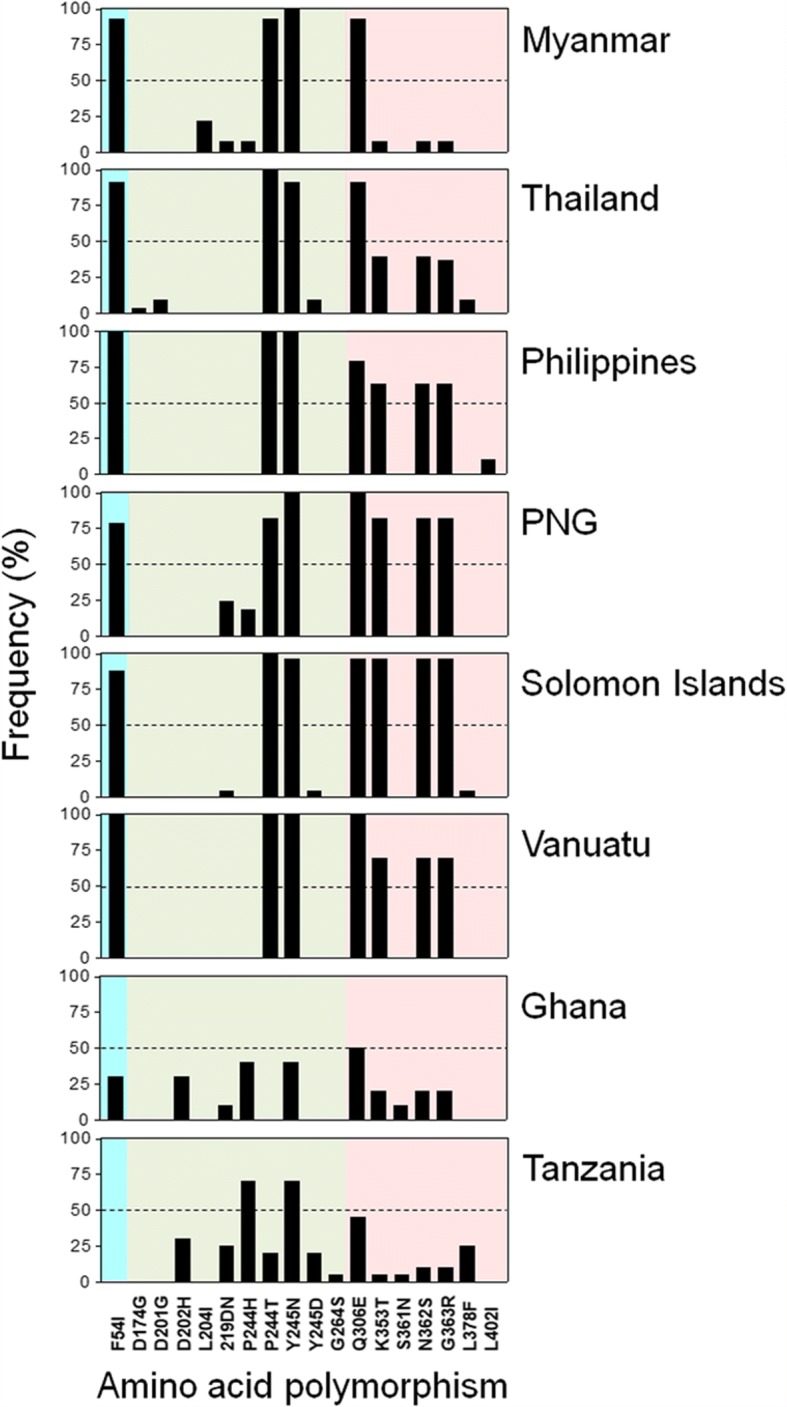
Comparison of amino acid polymorphisms in RO33 allele types of PfMSP-1_42_ among global *Plasmodium falciparum* isolates. Positions and frequencies of amino acid changes found in RO33 allele types of PfMSP-1_42_ in global *P. falciparum* isolates are compared. Blocks are indicated by different colors: block 15 (sky blue), block 16 (green), block 17 (pink). *Abbreviation*: PNG, Papua New Guinea

The pattern of amino acid polymorphisms in RO33 allele types of global PfMSP-1_42_ was also analysed (Fig. 4). A total of 18 amino acid polymorphisms were identified in global PfMSP-1_42_. The most notable amino acid changes that differed among global RO33 allele types of PfMSP-1_42_ were F54I, P244T, Y245N and Q306E. These amino acid changes were detected in all Asian and Pacific PfMSP-1_42_ analysed in this study with high frequencies up to 78%. However, they were less frequent in African PfMSP-1_42_. The three amino acid changes, K353T, N362S and G363R, were also detected with high frequencies in Asian and Pacific PfMSP-1_42_, but not in African PfMSP-1_42_. Unlike other Asian or Pacific PfMSP-1_42_, Myanmar PfMSP-1_42_ showed a lower level of polymorphisms for these three amino acids. Meanwhile, African PfMSP-1_42_ had a higher frequency of amino acid polymorphisms at P244H compared to Asian or Pacific PfMSP-1_42_. D202H and S361N were only identified in African PfMSP-1_42_, although their frequencies were not high.

### Nucleotide diversity and natural selection of Myanmar PfMSP-1_42_

Nucleotide sequence analysis was performed to determine nucleotide diversity and genetic differentiation of Myanmar PfMSP-1_42_. The *K-*values for MAD20, K1 and RO33 allele types of Myanmar PfMSP-1_42_ were 4.300, 1.431 and 1.506, respectively, with the highest *K*-value at block 16 for all three allele types (Table 1). In MAD20 allele types, overall haplotype diversity (Hd) and nucleotide diversity (π) for 37 Myanmar PfMSP-1_42_ sequences were 0.902 ± 0.040 and 0.00351 ± 0.00033, respectively. The π value for each block was 0.00313 ± 0.00049 for block 15, 0.00272 ± 0.00035 for block 16 and 0.00533 ± 0.00062 for block 17. The Hd for 18 K1 allele types of Myanmar PfMSP-1_42_ was 0.765 ± 0.080, and the π was 0.00120 ± 0.00026. When analysing the π value for each block, the highest value was predicted at block 15 (0.00214 ± 0.00037).

**Table 1:** DNA sequence polymorphism and test of neutrality for Myanmar PfMSP-1_42_

Allele type/ Fragment	Segregating sites	Singleton variable sites	Parsimony informative sites	Total no. of mutations	*K*	H	Hd ± SD	π ± SD	dN-dS	Tajima’s D (*P*)	Fu & Li’s D (*P*)	Fu & Li’s F (*P*)
MAD20												
Block 15	2	0	2	2	0.604	4	0.536 ± 0.066	0.00313 ± 0.00049		0.50117 (*P*>0.10)	0.78020 (*P*>0.10)	0.81005 (*P*>0.10)
Block 16	10	6	4	10	1.880	8	0.769 ± 0.043	0.00272 ± 0.00035		-0.65200 (*P*>0.10)	-2.01692 (*P*>0.10)	-1.86022 (*P*>0.10)
Block 17	7	2	5	7	1.817	6	0.632 ± 0.072	0.00533 ± 0.00062		0.23510 (*P*>0.10)	-0.20232 (*P*>0.10)	-0.07845 (*P*>0.10)
Full	19	8	11	19	4.300	21	0.902 ± 0.040	0.00351 ± 0.00033	0.0028	-0.18385 (*P*>0.10)	-1.6252 (*P*>0.10)	-0.99150 (*P*>0.10)
K1												
Block 15	1	0	1	1	0.471	2	0.471 ± 0.082	0.00214 ± 0.00037		1.16615 (*P*>1.10)	0.66689 (*P*>0.10)	0.91015 (*P*>0.10)
Block 16	4	2	2	4	0.641	4	0.477 ± 0.134	0.00102 ± 0.00037		-1.34736 (*P*>0.10)	-0.70114 (*P*>0.10)	-1.00781 (*P*>0.10)
Block 17	2	1	1	2	0.320	3	0.307 ± 0.132	0.00093 ± 0.00042		-1.09629 (*P*>0.10)	-0.55220 (*P*>0.10)	-0.79776 (*P*>0.10)
Full	7	3	4	7	1.431	6	0.765 ± 0.080	0.00120 ± 0.00026	−0.0012	-1.00674 (*P*>0.10)	-0.51291 (*P*>0.10)	-0.74949 (*P*>0.10)
RO33												
Block 15	1	1	0	1	0.143	2	0.143 ± 0.119	0.00074 ± 0.00062		-1.15524 (*P*>0.10)	-1.39749 (*P*>0.10)	-1.52388 (*P*>0.10)
Block 16	4	3	1	4	0.791	4	0.571 ± 0.132	0.00115 ± 0.00039		-1.22200 (*P*>0.10)	-1.41428 (*P*>0.10)	-1.55406 (*P*>0.10)
Block 17	4	4	8	4	0.571	3	0.275 ± 0.148	0.00168 ± 0.00108		-1.79759 (*P*>0.10)	-2.27380 (*P*>0.10)	-2.44883 (*P*>0.10)
Full	9	8	1	9	1.506	6	0.736 ± 0.109	0.00123 ± 0.00041	0.0015	-1.79616 (*P*<0.05)	-2.26934 (*P*<0.05)	-2.45049 (*P*<0.05)

*Abbreviations*: *K* average number of pairwise nucleotide differences, *H* number of haplotypes, *Hd* haplotype diversity, *π* observed average pairwise nucleotide diversity, *dN* rate of non-synonymous mutations, *dS* rate of synonymous mutations

The Hd and π values for 14 RO33 allele types of Myanmar PfMSP-1_42_ were 0.736 ± 0.109 and 0.00123 ± 0.00041, respectively. The highest π value for RO33 allele type was identified at block 17 (0.00168 ± 0.00108). The dN-dS value was also estimated to examine whether natural selection contributed to the genetic diversity in Myanmar PfMSP-1_42_. The dN-dS values for MAD20 and RO33 allele types were 0.0028 and 0.0015, respectively, implying that positive natural selection might have occurred in these sequences. Meanwhile, dN-dS value for K1 allele type was -0.0012, suggesting that negative natural selection might have affected the allele type, but this value was not significant (Table 1). To further analyse the occurrence of natural selection in Myanmar PfMSP-1_42_ population, Tajima’s D values were calculated. Estimated Tajima’s D values for MAD20, K1 and RO33 allele types were all negative: -0.18385 (*P* > 0.10) for MAD20 allele types, -1.00674 (*P* > 0.10) for K1 allele types and -1.79616 (*P* < 0.05) for RO33 allele types. These results suggest that all three allele types of Myanmar PfMSP-1_42_ are under population size expansion and/or purifying selection pressure. Fu and Li’s D and F values for all three allele types of Myanmar PfMSP-1_42_ were also negative (Table 1).

### Nucleotide diversities in global PfMSP-1_42_

**Fig. 5:**
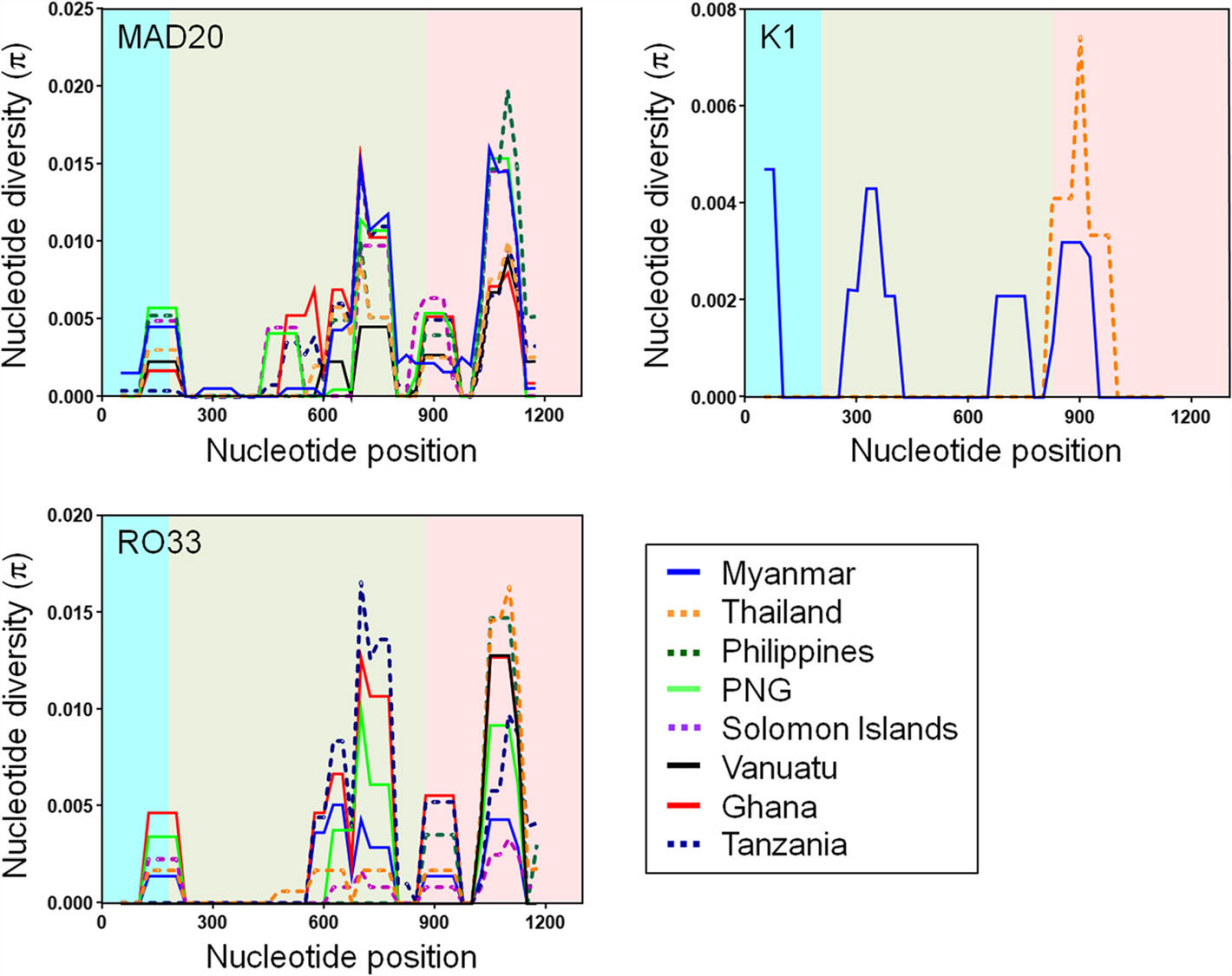
Nucleotide diversity of PfMSP-1_42_ in global *P. falciparum* isolates. Sliding window plot analysis illustrates nucleotide diversity (π) values in global PfMSP-1_42_ sequences analysed in this study. A window size of 100 bp and a step size of 25 bp were used. Blocks are indicated by different colors: block 15 (sky blue), block 16 (green), block 17 (pink). *Abbreviation*: PNG, Papua New Guinea

Nucleotide diversity of global PfMSP-1_42_ was analysed to understand the overall pattern of nucleotide diversity in global PfMSP-1_42_. Among PfMSP-1_42_ sequences from global isolates, including Myanmar, Thailand, Philippines, PNG, Solomon Islands, Vanuatu, Ghana and Tanzania, only 18 sequences of Myanmar isolates and 12 sequences of Thailand isolates were grouped to K1 allele types. Other sequences belonged to MAD20 or RO33 allele types. Values for nucleotide diversity (π) differed by allele types and isolates with different geographical origins (Table 2). Among MAD20 allele types of global PfMSP-1_42_, African PfMSP-1_42_ showed higher haplotype diversity (Hd) values than those from other geographical origins. Values of π for full-length PfMSP-1_42_ ranged from 0.00168 to 0.00351 in these isolates. However, all isolates showed similar patterns with the highest π values at block 17. Sliding window plot analysis of π also showed that values observed among global PfMSP-1_42_ sequences peaked at blocks 16 and 17, although the overall pattern was slightly distinct throughout sequences (Fig. 5). In the cases of K1 allele types, Myanmar and Thailand PfMSP-1_42_ had different patterns of π values for each block. Myanmar PfMSP-1_42_ showed nucleotide diversities in blocks 15 to 17, although their π values were not high. Meanwhile, Thailand PfMSP-1_42_ sequences were tightly conserved at blocks 15 and 16 (Table 2; Fig. 5). Similar patterns of dissimilar nucleotide diversity were also identified for RO33 allele types of global PfMSP-1_42_. Unlike PfMSP-1_42_ from Myanmar, Thailand, PNG, Solomon Islands and Ghana, PfMSP-1_42_ from Philippines, Vanuatu and Tanzania did not show nucleotide diversities at block 15 and/or block 16 (Table 2; Fig. 5). The highest value of π was found at block 17 for all isolates, ranging between 0.00122–0.00593. Based on the results of the full-length PfMSP-1_42_ analysis, African PfMSP-1_42_ had higher π values than PfMSP-1_42_ from other geographical isolates.

**Table 2:** Genetic polymorphisms and tests of neutrality for global PfMSP-1_42_

Allele type/ Country (No. of sequences)	*K*	H	Hd ± SD	π ± SD				dN-dS	Tajima’s D (*P*-value)			
				Block15	Block16	Block17	Full		Block15	Block16	Block17	Full
MAD20												
Myanmar (*n* = 37)	4.300	21	0.902 ± 0.040	0.00313 ± 0.00049	0.00272 ± 0.00035	0.00533 ± 0.00062	0.00351 ± 0.00033	0.0208	0.50117 (*P*>0.10)	-0.65200 (*P*>0.10)	0.23510 (*P*>0.10)	-0.18385 (*P*>0.10)
Thailand (*n* = 29)	2.620	6	0.473 ± 0.110	0.00153 ± 0.00048	0.00156 ± 0.00050	0.00364 ± 0.00094	0.00214 ± 0.00055	0.0027	0.25334 (*P*>0.10)	-0.42016 (*P*>0.10)	-0.53026 (*P*>0.10)	-0.46786 (*P*>0.10)
Philippines (*n* = 24)	3.880	5	0.652 ± 0.081	0.00268 ± 0.00018	0.00144 ± 0.00014	0.00694 ± 0.00082	0.00317 ± 0.00033	0.0040	1.57336 (*P*>0.10)	1.89198 (0.05<*P*<0.10)	2.22277 (*P*<0.05)	2.60308 (*P*<0.01)
PNG (*n* = 44)	4.098	7	0.753 ± 0.034	0.00263 ± 0.00011	0.00220 ± 0.00013	0.00607 ± 0.00029	0.00335 ± 0.00011	0.0035	1.67820 (*P*>0.10)	0.80323 *P*>0.10	1.99671 (0.05<*P*<0.10)	1.84977 (0.05<*P*<0.10)
SI (*n* = 17)	3.985	4	0.596 ± 0.099	0.00251 ± 0.00041	0.00205 ± 0.00036	0.00612 ± 0.00097	0.00326 ± 0.00052	0.0014	1.23804 (*P*>0.10)	1.67363 (*P*>0.10)	1.32924 (*P*>0.10)	1.79153 (0.05<P<0.10)
Vanuatu (*n* = 48)	2.052	3	0.228 ± 0.075	0.00116 ± 0.07200	0.00097 ± 0.00031	0.00339 ± 0.00110	0.00168 ± 0.00054	0.0019	-0.01160 (*P*>0.10)	-0.01781 (*P*>0.10)	-0.36701 (*P*>0.10)	-0.25695 (*P*>0.10)
Ghana (*n* = 23)	3.715	14	0.925 ± 0.041	0.00086 ± 0.00051	0.00324 ± 0.00019	0.00385 ± 0.00097	0.00304 ± 0.00029	0.0038	-0.66215 (*P*>0.10)	1.91092 (0.05<*P*<0.10)	-0.09195 (*P*>0.10)	0.84400 (*P*>0.10)
Tanzania (*n* = 51)	3.585	29	0.958 ± 0.014	0.00041 ± 0.00027	0.00296 ± 0.00028	0.00430 ± 0.00060	0.00293 ± 0.00023	0.0035	-1.46227 (*P*>0.10)	0.05855 (*P*>0.10)	0.25010 (*P*>0.10)	-0.15863 (*P*>0.10)
K1												
Myanmar (*n* = 18)	1.431	6	0.765 ± 0.080	0.00214 ± 0.00037	0.00102 ± 0.00037	0.00093 ± 0.00042	0.00120 ± 0.00026	-0.0012	1.16615 (*P*>1.10)	-1.34736 (*P*>0.10)	-1.09629 (*P*>0.10)	-1.00674 (*P*>0.10)
Thailand (*n* = 12)	0.742	3	0.530 ± 0.136	0	0	0.00215 ± 0.00081	0.00062 ± 0.00023	0.0008	0	0	-0.82879 (*P*>0.10)	-0.82879 (*P*>0.10)
RO33												
Myanmar (*n* = 14)	1.506	6	0.736 ± 0.109	0.00074 ± 0.00062	0.00115 ± 0.00039	0.00168 ± 0.00108	0.00123 ± 0.00041	0.0015	-1.15524 (*P*>0.10)	-1.22200 (*P*>0.10)	-1.79759 (*P*>0.10)	-1.79616 (*P*>0.10)
Thailand (*n* = 33)	2.375	6	0.680 ± 0.060	0.00088 ± 0.00043	0.00058 ± 0.00025	0.00529 ± 0.00055	0.00195 ± 0.00028	0.0024	-0.46603 (*P*>0.10)	-1.04700 (*P*>0.10)	1.23456 (*P*>0.10)	0.21599 (*P*>0.10)
Philippines (*n* = 19)	2.023	4	0.678 ± 0.088	0	0	0.00593 ± 0.00083	0.00166 ± 0.00023	0.0021	0	0	1.28875 (*P*>0.10)	1.28875 (*P*>0.10)
PNG (*n* = 33)	2.257	4	0.447 ± 0.093	0.00179 ± 0.00043	0.00144 ± 0.00030	0.00270 ± 0.00076	0.00185 ± 0.00047	0.0023	0.60324 (*P*>0.10)	0.78518 (*P*>0.10)	0.56203 (*P*>0.10)	0.89252 (*P*>0.10)
SI (*n* = 24)	0.811	3	0.236 ± 0.109	0.00118 ± 0.00053	0.00024 ± 0.00022	0.00122 ± 0.00110	0.00066 ± 0.00048	0.0008	-0.24844 (*P*>0.10)	-1.51469 (*P*>0.10)	-1.99611 (*P*<0.05)	-1.99292 (*P*<0.05)
Vanuatu (*n* = 43)	1.278	2	0.432 ± 0.057	0	0	0.00375 ± 0.00051	0.00106 ± 0.00014	0.0013	0	0	0.90772 (*P*>0.10)	0.90772 (*P*>0.10)
Ghana (*n* = 10)	4.022	6	0.844 ± 0.103	0.00242 ± 0.00068	0.00252 ± 0.00056	0.00534 ± 0.00144	0.00329 ± 0.00054	0.0041	0.81980 (*P*>0.10)	0.86725 (*P*>0.10)	0.12431 (*P*>0.10)	0.61111 (*P*>0.10)
Tanzania (*n* = 20)	3.694	13	0.953 ± 0.028	0	0.00250 ± 0.00030	0.00437 ± 0.00086	0.00303 ± 0.00032	0.0038	0	0.37812 (*P*>0.10)	-0.37024 (*P*>0.10)	0.03013 (*P*>0.10)

*Abbreviations*: *K* average number of pairwise nucleotide differences, *Hd* haplotype diversity, *π* observed average pairwise nucleotide diversity, *dN* rate of non-synonymous mutations, *dS* rate of synonymous mutations, *PNG* Papua New Guinea, *SI* Solomon Islands

### Natural selection and recombinant events in global PfMSP-1_42_

**Fig. 6:**
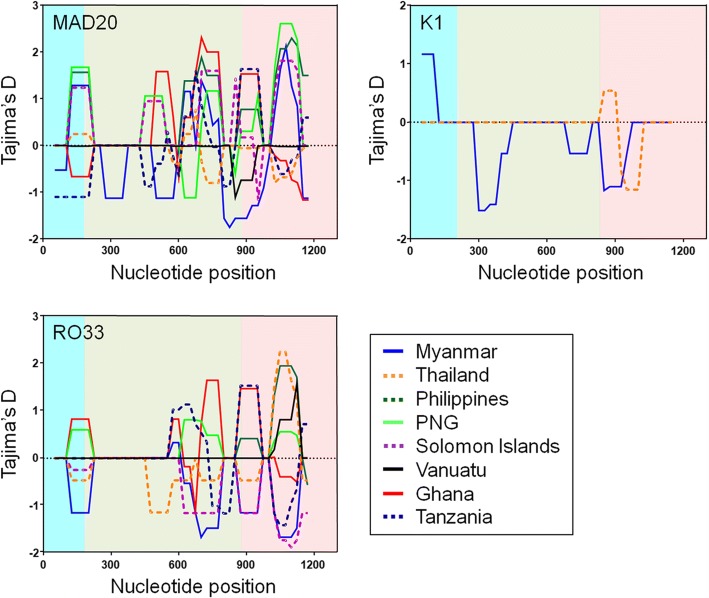
Tajima’s D of PfMSP-1_42_ in global *P. falciparum* isolates. Sliding window plot analysis of Tajima’s D was performed for global PfMSP-1_42_ sequences. A window size of 100 bp and a step size of 25 bp were used. Blocks are indicated by different colors: block 15 (sky blue), block 16 (green), block 17 (pink). *Abbreviation*: PNG, Papua New Guinea

To analyse the effect of natural selection on genetic diversity of global PfMSP-1_42_, the value of dN-dS was estimated. The dN-dS values for full-length PfMSP-1_42_ in global isolates were all positive except for K1 allele types in Myanmar PfMSP-1_42_, suggesting that balancing selection might have occurred in PfMSP-1_42_ of global *P. falciparum* population (Table 2). Global PfMSP-1_42_ sequences showed positive or negative Tajima’s D values (Table 2). The value for each block in global PfMSP-1_42_ also differed by allele types and geographical origins. A sliding window plot analysis also revealed that PfMSP-1_42_ from different geographical regions showed different patterns for Tajima’s D across the PfMSP-1_42_ gene (Fig. 6). The minimum number of recombination events between adjacent polymorphic sites (Rm) for global PfMSP-1_42_ was analysed. Recombination parameters were different among global PfMSP-1_42_ by allele types and isolates from different countries (Table 3). The higher value of the recombination parameters (Rm and R) indicate that high meiotic recombination is taking place between the sites generating genetic diversity in the gene. The highest R-values were predicted for African PfMSP-1_42_ (Ghana and Tanzania). The Rm values for MAD20 allele types of Ghana and Tanzania were 4 and 4, respectively. The values for RO33 allele types in Ghana and Tanzania were 2 and 3, respectively. The values of R (both between adjacent sites and for the entire domain) were also highest in Tanzania (0.0444 and 54.3 for MAD20 allele types and 0.0439 and 53.5 for RO33 allele types), followed by Ghana (0.0340 and 41.6 for MAD20 allele types and 0.0187 and 22.8 for RO33 allele types).

**Table 3:** Recombination events in global PfMSP-1_42_. The Ra, Rb and Rm were estimated excluding the sites containing alignment gaps or those segregating for three nucleotides

Allele type	Country (No. of sequences)	Ra	Rb	Rm
MAD20	Myanmar (*n* = 37)	0.0134	16.4	4
	Thailand (*n* = 29)	0	0.001	1
	Philippines (*n* = 24)	0.0004	0.5	0
	PNG (*n* = 44)	0.0028	3.4	2
	SI (*n* = 17)	0	0.001	0
	Vanuatu (*n* = 48)	0	0.001	0
	Ghana (*n* = 23)	0.0340	41.6	4
	Tanzania (*n* = 51)	0.0444	54.3	4
K1	Myanmar (*n* = 18)	0.0363	43.3	0
	Thailand (*n* = 12)	0.0083	9.9	0
RO33	Myanmar (*n* = 14)	0.0035	4.3	0
	Thailand (*n* = 33)	0.0028	3.4	1
	Philippines (*n* = 19)	0.0028	3.4	0
	PNG (*n* = 33)	0	0.001	1
	SI (*n* = 24)	0	0.001	0
	Vanuatu (*n* = 43)	0	0.001	0
	Ghana (*n* = 10)	0.0187	22.8	2
	Tanzania (*n* = 20)	0.0439	53.5	3

*Abbreviations*: *Ra* recombination parameter between adjacent sites, *Rb* recombination parameter for entire gene, *Rm* minimum number of recombination events between adjacent sites, *PNG* Papua New Guinea, *SI* Solomon Islands

Meanwhile, no recombination event was identified in PfMSP-1_42_ from the Solomon Islands or Vanuatu. Recombination parameters identified in PfMSP-1_42_ were not high, and they differed by geographical population, but a possible recombination event might have occurred in global PfMSP-1_42_.

### Haplotype network analysis

**Fig. 7:**
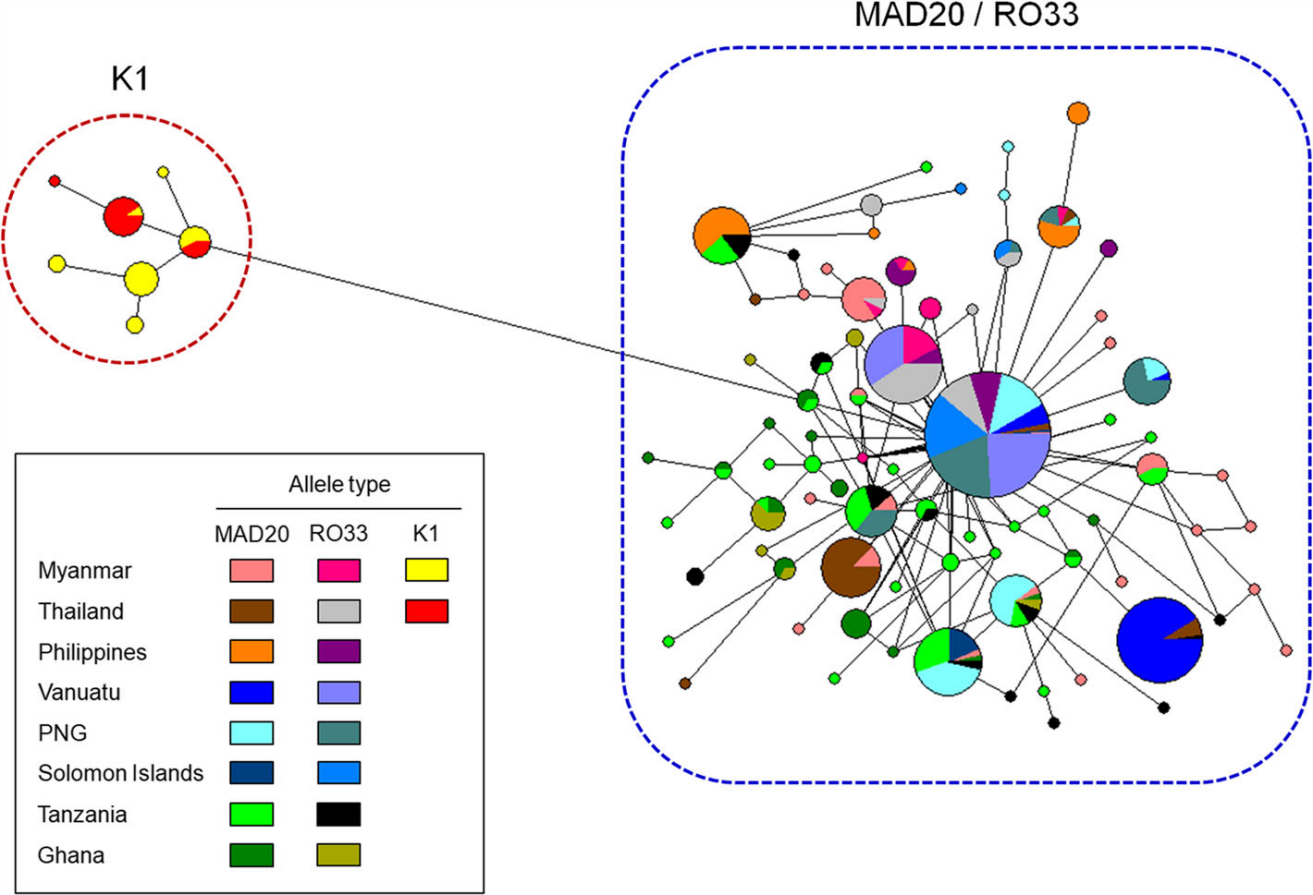
Network analysis of global PfMSP-1_42_ haplotypes. Haplotype network was constructed using the program NETWORK version 5.0.0.3 with the Median Joining algorithm. A total of 499 PfMSP-1_42_ sequences were analysed. The size of each circle reflects the richness of the haplotype, which each color showing the geographical origin and allele type of the isolates. The lengths of the lines connecting the circles, measured from their centres, is proportional to the number of base pair substitutions separating the haplotypes. Color of each node indicates different country and allele type

Haplotype network analysis of PfMSP-1_42_ haplotypes from global *P. falciparum* populations showed a dense network with extremely complex relationships (Fig. 7). A total of 86 distinct haplotypes were identified in 499 PfMSP-1_42_ sequences analysed, of which 70.9% (61/86) was a singleton. Haplotype prevalence ranged between 0.2–24.2%. K1 allele type formed a separated group from MAD20 and RO33 allele types. Meanwhile, MAD20 and RO33 clustered into one group with mixed patterns probably due to their high sequence similarities. Interestingly, the rate of a singleton was high in African PfMSP-1_42_, MAD20 and RO33 allele types, which support a high level of genetic diversity in the African PfMSP-1_42_ population.

## Discussion

The population structure of malaria parasites plays a vital role in maintaining natural immunity against malaria. Several factors including transmission intensity, parasite recombination rate, antimalarial drug resistance status, and effective malaria control measures can affect the population diversity of parasites. In this respect, population structure analysis of malaria parasites is essential to understand the genetic nature and evolutionary aspect of the parasites, especially for vaccine candidate antigens or drug resistance genes. PfMSP-1_42_ is the target of immune protection. Therefore, PfMSP-1_42_ has been recognised as a promising candidate antigen for blood stage vaccine development [17, 20–23]. Unlike other regions of PfMSP-1, PfMSP-1_42_ has been considered as genetically stable among *P. falciparum* isolates [26, 27]. This is one of the main reasons why it has been recognised as a potential vaccine candidate. However, only limited information is currently available on genetic structure, and natural selection of PfMSP-1_42_ in global *P. falciparum* isolates. In this study, comparative analysis of population structure and natural selection of PfMSP-1_42_ in Myanmar and global *P. falciparum* isolates was performed.

Myanmar and global PfMSP-1_42_ displayed limited levels of genetic diversity. However, non-negligible genetic diversity was also found. Thus, it is difficult to regard that PfMSP-1_42_ is genetically well conserved in global *P. falciparum* population. Genetic diversity of PfMSP-1 has been defined as unique, different allele types: MAD20, K1 and RO33 [26, 27]. Blocks 15, 16 and 17 of PfMSP-1 comprising PfMSP-1_42_ have been considered to be well conserved in *P. falciparum* population [26, 27, 47]. All three allele types of Myanmar and global PfMSP-1_42_ showed SNPs that were mainly identified in blocks 16 and 17. Overall distribution patterns and frequencies of amino acid changes found in Myanmar and global PfMSP-1_42_ analysed in this study were not identical between or among global PfMSP-1_42_. The implication of these substantial differences in amino acid polymorphisms among global PfMSP-1_42_ by geographical differentiation is currently unclear. Geographical isolation followed by population subdivision may affect genetic differences of PfMSP-1_42_ population in different geographical areas. Major amino acid changes found in global PfMSP-1_42_ were commonly detected among global PfMSP-1_42_, although their frequencies differed among and between populations and only a limited number of PfMSP-1_42_ sequences in each geographical area were analysed in this study. Thus, further examination of PfMSP-1_42_ nucleotide and amino acid variations in a more substantial number of global PfMSP-1_42_ sequences is needed to better understand the polymorphic nature of global PfMSP-1_42_.

Values of haplotype diversity (Hd) and nucleotide diversity (π) were higher in African PfMSP-1_42_ than those in Asian and Pacific PfMSP-1_42_, indicating that African PfMSP-1_42_ possessed higher levels of genetic diversities compared to Asian and Pacific PfMSP-1_42_. For both MAD20 and RO33 allele types, the highest π value was identified at block 17 in global PfMSP-1_42_, suggesting that block 17 had the highest genetic variations. Sliding window plot analysis of π across PfMSP-1_42_ revealed similar patterns of nucleotide diversity in global MAD20 and RO33 allele types of PfMSP-1_42_ analysed here, suggesting that global PfMSP-1_42_ might share similar nucleotide diversity across this gene. In the case of K1 allele types, different patterns of π were identified between Myanmar and Thailand PfMSP-1_42_. Nucleotide diversity in Thailand PfMSP-1_42_ was identified only at block 17 while Myanmar PfMSP-1_42_ showed nucleotide diversity at blocks 15, 16 and 17, albeit π values for these regions were not high. Natural selection analysis of global PfMSP-1_42_ suggests that this region is likely to be under natural selection which may maintain or produce genetic diversity in PfMSP-1_42_ population. The dN-dS value for global PfMSP-1_42_ was positive, implying balancing selection might act in this gene. However, dN-dS value for Myanmar K1 allele types was negative (-0.0012), suggesting negative or purifying natural selection in this region. In the sliding window plot analysis of Tajima’s D, overall patterns of Tajima’s D values across full-length PfMSP-1_42_ showed highly complicated patterns that were distinct between and among global PfMSP-1_42_. These results suggested that global PfMSP-1_42_ was under a complicated influence of natural selection, in which either positive natural selection or purifying selection might have occurred in the population depending on allele types and geographical origins. Possible recombination events in global PfMSP-1_42_ were also predicted, although values were not high. Higher values of recombination events were found in African PfMSP-1_42_ than those in PfMSP-1_42_ from other geographical areas, suggesting that African PfMSP-1_42_ might be under more opportunity for inter- or intra-allelic recombination than other geographical PfMSP-1_42_ probably due to high multiclonal infection rate, subsequent cross-fertilisation and recombination in mosquitoes. These results collectively suggest that PfMSP-1_42_ is not highly conserved in global population as known to date and that complicated natural selection acts on this region. Recombination might also contribute to the genetic diversity of global PfMSP-1_42_, albeit recombination parameters were not high.

Of three blocks of PfMSP-1_42_, block 17 (referred to as PfMSP-1_19_) has attracted considerable attention since this region is functionally critical. PfMSP-1_19_ has two EGF-like motifs that play an important role in the attachment of the parasite to specific receptors on the surface of erythrocyte [6]. Antibodies to one of these two EGF-like motifs in PfMSP-1_19_ are strongly associated with resistance to both clinical malaria and high levels of parasitemia [48]. It has been proposed that PfMSP-1_19_ is conserved mainly between parasite strains with only a few amino acid substitutions [26, 27, 47]. However, results of this study suggested that block 17 also showed greater polymorphic patterns in global isolates than previously known, especially for MAD20 and RO33 allele types. A possible recombination event in this region was also predicted, in concordance with previous reports [26, 27, 47, 49]. SNPs in PfMSP-1_19_ might have evolved to evade the human immune response capable of blocking the parasite from invading erythrocytes [27, 48]. Polymorphic nature, possible natural selection and recombination events observed at block 17 in global isolates raise concern for reduced efficacy of vaccine using PfMSP-1_19_. Therefore, caution is needed when designing polyvalent vaccine constructs targeting PfMSP-1_19_. Comprehensive genetic analysis of global PfMSP-1_19_ is merited to understand the complexity of genetic makeup of this region in the global *P. falciparum* population.

## Conclusions

Comparative analysis of Myanmar PfMSP-1_42_ with global PfMSP-1_42_ revealed that global PfMSP-1_42_ displayed limited levels of genetic diversity in the *P. falciparum* population. However, results of this study also suggest that global PfMSP-1_42_ is not as genetically conserved as previously thought. Although patterns of major amino acid changes found in global PfMSP-1_42_ were relatively similar, their frequencies differed by allele types and geographical origins. Moreover, some amino acid changes were characteristic only in specific geographical origins. Global PfMSP-1_42_ was under the complicated influence of natural selection, in which either balancing selection or purifying selection might have occurred in the population depending on allelic types and geographical origins. Recombination might have also affected the genetic diversity of the global PfMSP-1_42_ population. These results collectively suggested that more concern would be necessary when designing vaccine based on PfMSP-1_42_ which has been considered to be well conserved in *P. falciparum* population. This study also warrants continuous monitoring of genetic analysis for PfMSP-1_42_ in global PfMSP-1_42_ to better understand the polymorphic nature and evolutionary aspect of PfMSP-1_42_ in the global *P. falciparum* population.

## Additional files

Additional file 1: **Figure S1.** Sequence analysis for MAD20 allele types of PfMSP-1_42_ in Myanmar *Plasmodium falciparum* isolates. Regions corresponding to blocks 15, 16 and 17 are marked with different colors. Dots represent identical residues compared to reference sequences. Underlines indicate regions of epidermal growth factor-like (EGF1 and EGF2) domains. Amino acid changes identified in at least one sequence of the corresponding haplotype are marked in red. Cysteine residues in block 17 are shown in blue with an underline. Total numbers of isolates for each haplotype are listed in the right panel. (TIF 3611 kb)

Additional file 2: **Figure S2.** Sequence analysis for K1 allele types of PfMSP-1_42_ in Myanmar *Plasmodium falciparum* isolates. Regions corresponding blocks 15, 16 and 17 are marked with different colors. Dots represent identical residues compared to reference sequences. Regions of epidermal growth factor-like (EGF1 and EGF2) domains are indicated by underlines. Amino acid changes identified in at least one sequence of the corresponding haplotype are marked in red. Cysteine residues in block 17 are shown in blue with an underline. Total numbers of isolates for each haplotype are listed in the right panel. (TIF 1834 kb)

Additional file 3: **Figure S3.** Sequence analysis for RO33 allele types of PfMSP-1_42_ in Myanmar *Plasmodium falciparum* isolates. Regions corresponding blocks 15, 16 and 17 are marked with different colors. Dots represent identical residues compared to reference sequences. Regions of epidermal growth factor-like (EGF1 and EGF2) domains are indicated by underlines. Amino acid changes identified in at least one sequence of the corresponding haplotype are marked in red. Cysteine residues in block 17 are shown in blue with an underline. Total numbers of isolates for each haplotype are listed in the right panel. (TIF 1791 kb)

## Abbreviations

dN: Rate of non-synonymous substitutions
dS: Rates of synonymous substitutions
H: Haplotypes
Hd: Haplotype diversity
*K*: Average number of pairwise nucleotide differences within the population
MSP-1: Merozoite surface protein-1
PCR: Polymerase chain reaction
*PfMSP-1*_*42*_: *C-terminal 42 kDa region* of merozoite surface protein-1 of *Plasmodium falciparum*
PNG: Papua New Guinea
Rm: Minimum number of recombination events between adjacent polymorphic sites
S: Segregating sites
π: Nucleotide diversity
