# Supplementary figures and images for: Genetic diversity of merozoite surface protein-1 C-terminal 42 kDa of Plasmodium falciparum (PfMSP-142) may be greater than previously known in global isolates
Source: Parasit Vectors. 2018 Aug 6;11:455. doi: 10.1186/s13071-018-3027-x (PMC6080494; doi:10.1186/s13071-018-3027-x)

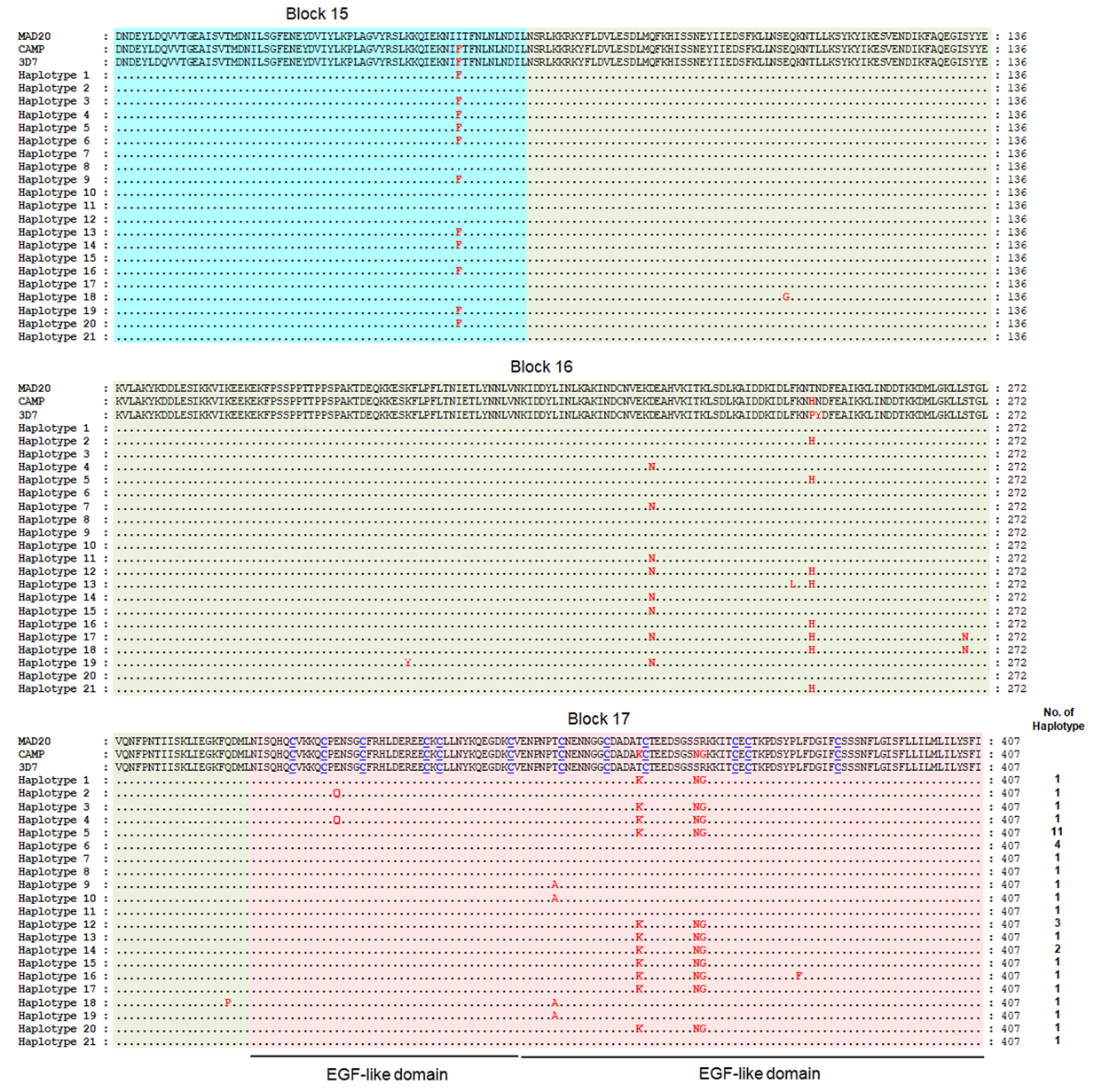

Supplement: Supplementary file 1 — Figure S1. Sequence analysis for MAD20 allele types of PfMSP-142 in Myanmar Plasmodium falciparum isolates. Regions corresponding to blocks 15, 16 and 17 are marked with different colors. Dots represent identical residues compared to reference sequences. Underlines indicate regions of epidermal growth factor-like (EGF1 and EGF2) domains. Amino acid changes identified in at least one sequence of the corresponding haplotype are marked in red. Cysteine residues in block 17 are shown in blue with an underline. Total numbers of isolates for each haplotype are listed in the right panel. (TIF 3611 kb) [file 13071_2018_3027_MOESM1_ESM.tif]

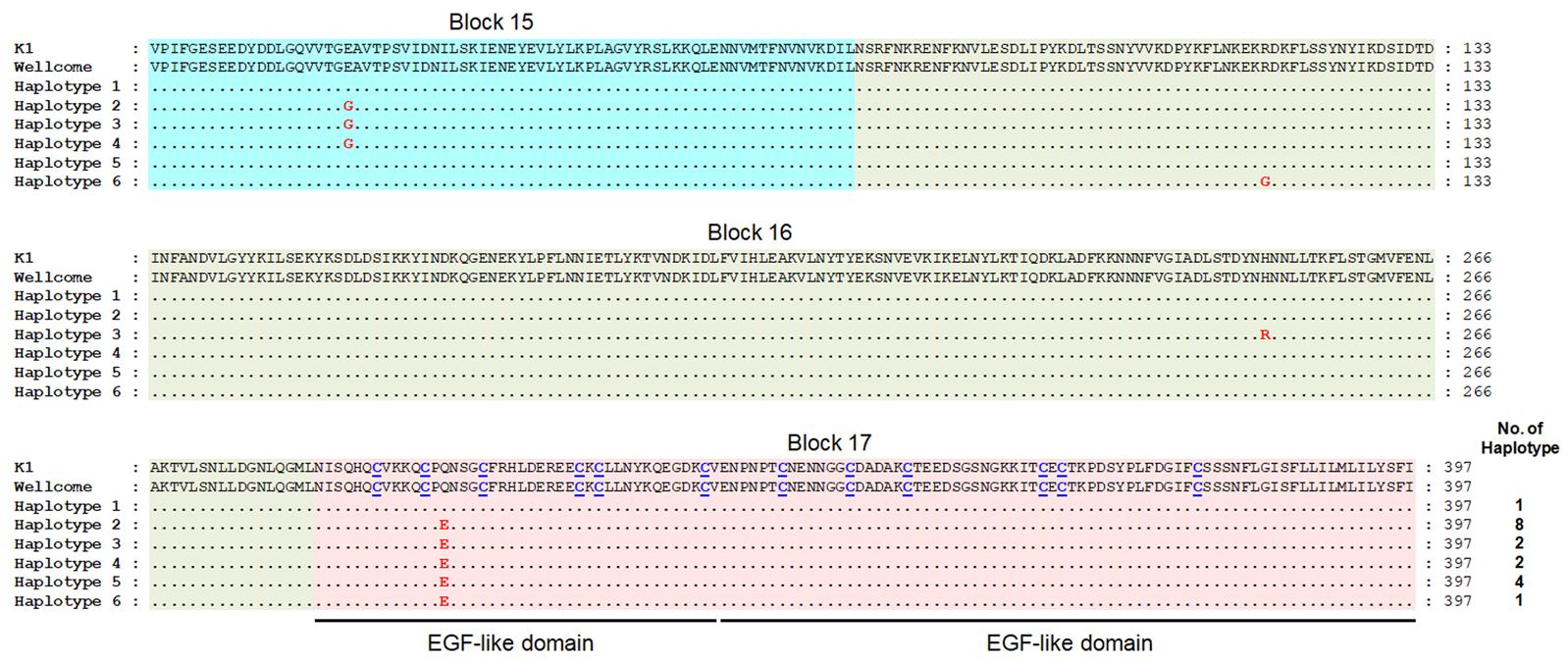

Supplement: Supplementary file 2 — Figure S2. Sequence analysis for K1 allele types of PfMSP-142 in Myanmar Plasmodium falciparum isolates. Regions corresponding blocks 15, 16 and 17 are marked with different colors. Dots represent identical residues compared to reference sequences. Regions of epidermal growth factor-like (EGF1 and EGF2) domains are indicated by underlines. Amino acid changes identified in at least one sequence of the corresponding haplotype are marked in red. Cysteine residues in block 17 are shown in blue with an underline. Total numbers of isolates for each haplotype are listed in the right panel. (TIF 1834 kb) [file 13071_2018_3027_MOESM2_ESM.tif]

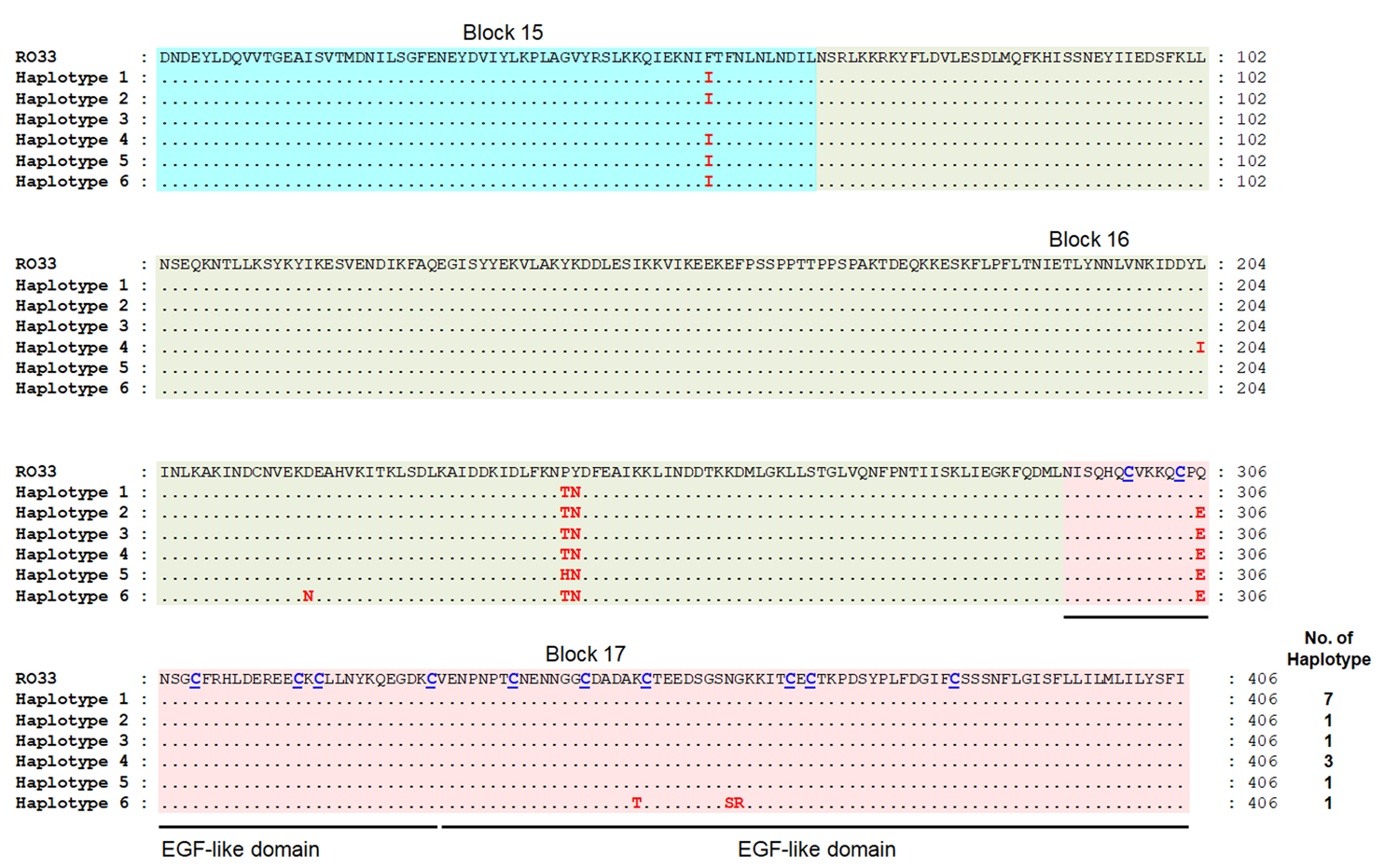

Supplement: Supplementary file 3 — Figure S3. Sequence analysis for RO33 allele types of PfMSP-142 in Myanmar Plasmodium falciparum isolates. Regions corresponding blocks 15, 16 and 17 are marked with different colors. Dots represent identical residues compared to reference sequences. Regions of epidermal growth factor-like (EGF1 and EGF2) domains are indicated by underlines. Amino acid changes identified in at least one sequence of the corresponding haplotype are marked in red. Cysteine residues in block 17 are shown in blue with an underline. Total numbers of isolates for each haplotype are listed in the right panel. (TIF 1791 kb) [file 13071_2018_3027_MOESM3_ESM.tif]
